# The relationship of perceived discrimination in healthcare and future falls among community-dwelling older persons from an English longitudinal cohort

**DOI:** 10.12688/f1000research.140302.1

**Published:** 2023-09-11

**Authors:** Felipe Alfonso Sandoval Garrido, Timothy Bolt, Yuta Taniguchi, Peter Lloyd-Sherlock

**Affiliations:** 1 Health Services Research Department, University of Tsukuba, Tsukuba, Ibaraki Prefecture, 3057583, Japan; 2 Faculty of Medicine, University of Tsukuba, Tsukuba, Ibaraki Prefecture, 3058573, Japan; 3 Faculty of Economics, Saitama University, Saitama, Saitama Prefecture, 3388570, Japan; 4 School of International Development, University of East Anglia, Norwich, England, NR4 7TJ, UK

**Keywords:** perceived discrimination, health services, falls, older persons, England

## Abstract

Background: The objective of this study is to examine the relation between the perceived discrimination suffered by older adults aged 60 and over during a healthcare encounter and its effects on the likelihood of falling 4 and 8 years later.

Methods: To identify discrimination, we used the English Longitudinal Study of Ageing (ELSA) data collected in 2010-2011 (wave 5) that asked respondents about feeling discriminated against by doctors or at hospitals in the past year. Falls were assessed by the question: “Have you fallen down in the last two years?” in subsequent waves. We performed longitudinal analyses using the 2014-2015 (wave 7) and 2018-2019 (wave 9) follow-ups. Multivariable logistic regression was used to estimate the odds ratios of falling.

Results: At baseline, 707 (15.1%) of all respondents experienced healthcare discrimination. Those suffering from discrimination in health care had 64% higher chances of falling 4 years later (odds ratio: 1.637, 95% confidence interval: 1.131-2.368) compared to those who did not, adjusting for age, sex, marital status, wealth, ethnicity, education levels, self-perceived health, depressive symptoms, and difficulties with basic and/or instrumental activities of daily living (ADL/IADL) and difficulties with walking. After 8 years, the effect was not statistically significant. Older age was the only significant detrimental factor at both 4 and 8 years.

Conclusions: Understanding discrimination in health care is important to enable safe and welcoming environments for the timely future use of services. These results remind us of the physical risk and the complex panorama of bio-psychosocial determinants involved in tackling discrimination over time.

## Introduction

As people age, they experience psychological, physical, and societal transformations that greatly affect their overall health and well-being. ^1^ Among these changes, falls emerge as a public health concern for older individuals.

The number of fatal falls that occur yearly is estimated to be 684,000, making falls the second greatest cause of unintentional injury-related fatalities behind traffic accidents. ^2^ In addition to mortality, falls contribute significantly to disability in older adults. Approximately 37.3 million falls yearly are severe enough to require medical attention worldwide. ^2^ Injuries resulting from falls, such as fractures or traumatic brain injuries, often lead to long-term disabilities. ^3^ In the world, around 172 million persons suffer from either short or long-term disabilities after falling. ^2^

The 2019 and latest Cochrane Review on falls, with data from over 25 countries, shows that one-third of community-dwelling older persons aged 65 and over fall each year. ^4^ In the United States, evidence shows that 10% of falls result in major injuries and 30% to 50% in minor ones. ^5^ Hip fractures occurred in around 1% of all falls, but 95% of hip fractures were caused by falls. ^5^ In the United Kingdom, the Public Health Outcomes Framework reports that between 2017-2018 falls led to approximately 220,160 hospital admissions for individuals aged 65 and over. Around two-thirds of these cases (146,665 cases) were patients aged 80 and above. ^6^ In the 2021/2022 period, the number of hospital admissions due to falls in people aged 65 and over had risen to 223,101 cases (about 2,100 per 100,000 persons). Among these, nearly 147,000 involved individuals were aged over 80. ^6^

Apart from the harm they can cause, falls also have consequences that negatively affect the well-being of older individuals. One common psychological response among those who have experienced a fall or are at risk is the fear of falling. ^7^ Following a fall, this fear leads to decreased independence and self-imposed limitations on activities and isolation from interactions. All these factors contribute to declining quality of life. ^8^

Understanding the reasons behind falls in adults is crucial in order to develop strategies for preventing them. ^4^ As individuals age, various physiological changes occur, including decreased bone density, muscle mass and strength. These changes can compromise balance, gait and overall physical stability, making older adults more susceptible to falling. ^9^ Diminished cardiovascular performance and degenerative neurological changes in older adults also contribute to this risk, leading to dizziness, fainting spells, slower reaction times, and impaired coordination. ^10^ Moreover, age-associated vision and hearing impairments negatively affect balance and spatial orientation, further increasing fall risk. ^11^

While these physiological factors leading to falls have traditionally been the focus, the importance of psychosocial factors cannot be overlooked. ^12^ Perceived discrimination in healthcare has emerged as a significant concern for older adults. Discrimination based on age, sex, race, or socioeconomic status can erode trust in healthcare providers, lead to avoidance of healthcare, and result in increased stress levels and poorer overall health outcomes ^13^^,^^14^ and thus contributing to the risk of falling.

A previous study explored the relationship of discrimination and falling using a representative national sample in Colombia. ^15^ Based on their previous research, the rationale is that traumatic events have an effect in the possibility of falling, ^16^ but there were no studies linking discrimination and falls, unlike other adverse events with a more direct connection. From a life course perspective, the argument is that severe stressors that continue overtime or are repeated could lead to other secondary stressors, following the stress proliferation theory. ^17^ In line with this, the physical and mental damage caused by feeling discriminated will have an indirect and future effect on falls. The idea of exploring the indirect effects of perceived discrimination has been reviewed before suggesting pathways to negative health outcomes. ^14^

However, besides the work of Reyes *et al*., ^15^ the role of perceived discrimination as a potential risk factor for falls remains largely unexplored, particularly in England where discrimination among older populations remains understudied. ^18^ The aim of the study is to grasp the relationship between feeling discriminated and the likelihood of falling in 2 subsequent points, measured at 4-year intervals.

Understanding the potential long-term effects of perceived discrimination on the risk of falling can help one develop multifaceted intervention strategies that incorporate physical and psychosocial components, enabling a more comprehensive approach to fall prevention and addressing the need for more inclusive, respectful, and patient-centred healthcare environments.

## Methods

### Study design

This study used a longitudinal design to examine the relationship between perceived discrimination during healthcare encounters and subsequent fall risks in older adults. The longitudinal nature of this research design allowed us to investigate changes in individuals’ risk of falls over a period following experiences of discrimination, providing insights into the temporal dynamics of this association.

### Participants and sampling

The data used in this research was obtained from the English Longitudinal Study of Ageing (ELSA) which is a study that follows adults aged 50 and older living in England. ELSA aims to gather data over time on different fields such as mental health, disabilities, biological indicators of diseases, economic situation, social engagement, wellbeing and more. ^19^ Participants provided consent at the start of the longitudinal study as part of the Health Survey for England (HSE). Later, at the end of each ELSA wave, they are asked again to confirm or amend their consent.

This study focused on ELSA participants during the 2010-2011 wave of data collection (wave 5). Wave 5 is the only time where questions about discriminations have been included, as of early 2023. We also used the data from periods 2014-2015 (wave 7) and 2018-2019 (wave 9) to detect falls in these two subsequent periods.

**Figure 1.**
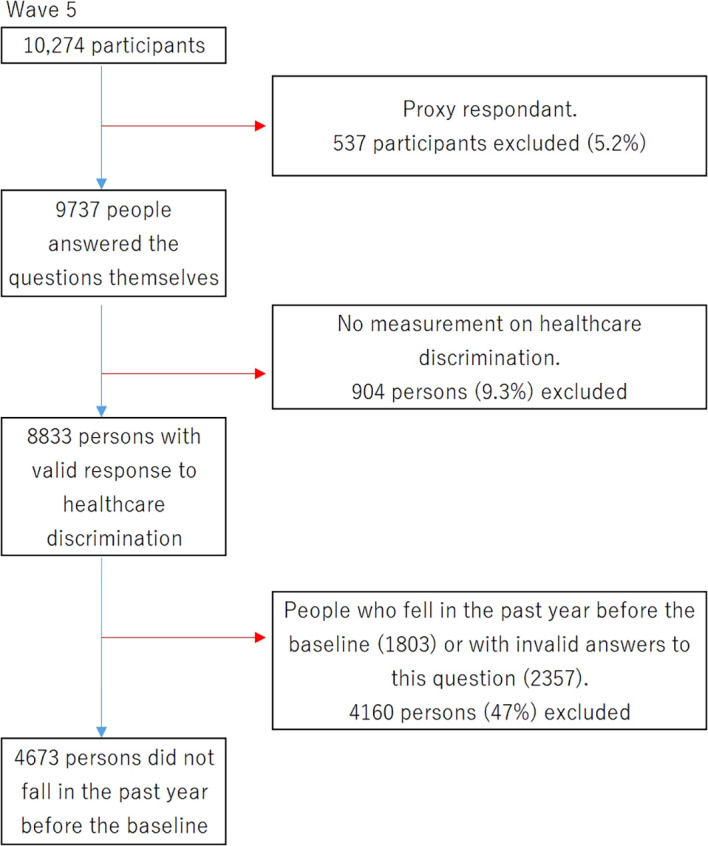
Inclusion and exclusion criteria for the sample of participants.

The flow of participants through the study is depicted in Figure 1, illustrating the inclusion and exclusion criteria for the sample. In wave 5, a total of 10,274 participants were initially enrolled.

Due to the subjective nature of feeling discriminated, which takes into account unique perceptions, feelings, and experiences, proxy respondents were excluded from the study. Thus, 537 participants (5.2%) were dropped from the research because they needed a proxy respondent to reply to the questions. This means that 9737 individuals, or 94.8%, were able to answer the questions on their own. Additionally, 904 individuals (9.3% of the remaining sample) were subsequently eliminated as a result of lacking information about healthcare discrimination. This led to a sample size of 8,833 individuals who provided valid responses regarding healthcare discrimination. An additional exclusion criterion was applied to individuals who reported falling in the past year before the baseline (excluding 1803 participants) or provided invalid answers to this question (2347 excluded participants), leaving a total of 4,673 participants who confirmed not experiencing a fall in the year prior to the baseline assessment.

Overall, the study included 4,673 participants who did not fall in the past year, had valid responses regarding healthcare discrimination, and provided self-reported answers to the study questions.

### Measures

Perceived discrimination

Perceived discrimination was identified using responses to questions in the ELSA 2010-2011 period (wave 5) about participants’ experiences of feeling discriminated by doctors or at hospitals in the past year by asking the frequency as follows:

In your day-to-day life, how often have any of the following things happened to you?

You receive poorer service or treatment than other people from doctors or hospitals.

1. Almost every day
2. At least once a week
3. A few times a month
4. A few times a year
5. Less than once a year
6. Never

As described in Figure 1, most participants answered that they have never felt discriminated. Due to this skewness, for our preliminary descriptive statistics we decided on a dichotomous variable that will separate the observations into those who never felt discriminated and those who felt discriminated at least once (options 1-5 vs 6). Later, based on our sensitivity analyses, for the multivariable analysis we created a dichotomous variable that separates the participants into those who experienced healthcare discrimination more frequently (options 1 to 4), against those who experience it less frequently (options 5 and 6).

Experience of falling

Falls were assessed using responses to the question, “Since we last talked to you on [date of last interview] have you fallen down in the last two years (for any reason)?” Possible answers were yes or no. This question, at baseline wave 5, was used as an exclusion criterion, to focus on the participants who had not fallen in the past two years, to isolate the effect of discrimination and minimize possible bias in dealing with a recurrent faller. In subsequent waves 7 and 9, this question was used as the main outcome variable.

Covariates

We controlled for several covariates that were organized into a demographic, socioeconomic, and health factors.

Demographic factors included age and sex. Age was categorized into four groups: less than 60 years, 60-69 years, 70-79 years, and over 80 years. Sex was categorized as male or female. Lastly, ethnicity was grouped as either ‘White’ or ‘Non-white’.

The determination of socioeconomic status was done based on two metrics: the wealth of the household and the educational background. Wealth was assessed without taking pensions into account, segmented into five distinct categories - the first meaning the least wealthy and the fifth being the wealthiest. Education level was categorized as low (no educational qualifications or incomplete lower level), middle (NVQ1/CSE and NVQ2/GCE O level or equivalent to primary and secondary school), and high (NVQ3/GCE A level, higher education below a degree or NVQ4/NVQ5 degree or equivalent to the last 2 years or secondary -vocational- or tertiary education). We also controlled for current working status which was categorized as ‘working’ or ‘not working’. Marital status representing those ‘in a partnership’ (either married or cohabiting) and those ‘not in a partnership’.

We also adjusted for health variables. These included self-perceived physical health answering to the question “Would you say your health is…”. The options were “1 excellent, 2 very good, 3 good, 4 fair, or 5 poor?” dividing those who consider their health to be fair or poor against the rest. We also assessed walking difficulties, a strong predictor falls, by whether individuals reported difficulty walking a quarter of a mile (400 metres) (‘Yes’ or ‘No’), and mental health status, determined using the Center for Epidemiologic Studies Depression (CES-D) scale. This scale classified participants into those with ‘No depressive symptoms’ (score less than 4) and those with ‘Depressive symptoms’ (score of 4 or more). Finally, we also included an indicator of lost independence in the form of difficulties with ADL/IADL.

### Data source

This study used data collected in the 2010-2011 period (wave 5) of ELSA for identifying perceived discrimination and the 2014-2015 (wave 7) and 2018-2019 (wave 9) waves for fall incidents. These waves were chosen to allow for a lag period of 4 and 8 years between the reported experiences of discrimination and fall incidents, providing adequate temporal distance to observe potential long-term impacts. As of early 2023, wave 5 remains the only wave to include questions on discrimination. Ethical clearance was obtained from the University of Tsukuba on October 14, 2022, notification number 1817.

### Statistical analysis

Bivariate analyses were conducted to examine the associations between the variables at baseline using chi-square analyses with 95% confidence intervals. A *p*-value of less than 0.05 was considered statistically significant.

Multivariate logistic regression models were used to estimate the odds ratios of falling 4 and 8 years after experiencing discrimination. Models were adjusted for potential confounding variables including age, marital status, self-perceived health, depressive symptoms, wealth, ethnicity, education levels, and difficulties walking, and with Activities of Daily Living (ADL) and Instrumental Activities of Daily Living (IADL). This approach allowed us to isolate the effect of perceived discrimination on the likelihood of falls while accounting for known demographic, health, and socioeconomic factors leading to falling. Statistical significance was set at *p* < 0.05. All analyses were performed using SPSS statistical software, version 29.0.1.0.

## Results

### Demographic characteristics and perceived discrimination in healthcare

In this study of 4673 individuals, the baseline sociodemographic characteristics, as well as economic and health factors, were analysed in relation to perceived age discrimination in healthcare settings. The results shown in Table 1 revealed that, out of the 4673 individuals, 707 perceived to be discriminated. Associations between perceived age discrimination and sociodemographic items were explored using the Pearson’s Chi-square statistic, with significance set at a *p*-value less than 0.05 (95% confidence interval). The variables are discussed in the following categories: sociodemographic, economic, and health factors.

### Sociodemographic factors

Age and education level emerged as significant sociodemographic factors associated with perceived discrimination ( *p*=0.023 and *p*=0.001, respectively). Younger participants (60-69) reported more discrimination than their older counterparts. Similarly, individuals with higher educational qualifications were more likely to report discrimination than those with middle or low levels of education. However, gender and marital status did not show significant associations with perceived discrimination (p=0.603 and p=0.161, respectively). Similarly, no significant difference was observed in the perceived discrimination reports between non-white and white participants ( *p*=0.201).

### Economic factors

In terms of economic factors, wealth quintiles based on non-pension wealth were not significantly associated with perceived discrimination ( *p*=0.776). This indicates that the level of wealth does not significantly influence the perception of age discrimination in healthcare settings in this sample.

### Health factors

The health-related factors were the most significantly associated with perceived discrimination. Perceived health status, depressive symptoms as measured by the CES-D scale, difficulties with ADL/IADLs, and the ability to walk a quarter mile (400 metres) unaided were all significantly associated with perceived discrimination ( *p*<0.001, *p*<0.001, *p*<0.001, and *p*=0.025, respectively). This means that participants who rated their health as fair or poor, those who exhibited depressive symptoms, those with difficulties in ADL/IADLs, and those who had difficulty walking unaided reported higher levels of discrimination. These findings indicate that individuals with poorer health status or limitations in functional ability perceive higher levels of age discrimination in healthcare settings. Full details can be found in Table 1 below.

**Table 1.** Associations between healthcare discrimination and sociodemographics at baseline (wave 5) (n=4673).

Variables	No Perceived Discrimination at a Healthcare Setting (n=3966)		Perceived Discrimination at a Healthcare Setting (n=707)		p-value
	n	%	n	%	
Age groups					0.023
60-69	2132	83.6%	418	16.4%	
70-79	1379	86.7%	211	13.3%	
Over 80	455	85.4%	78	14.6%	
Sex					0.603
Male	1882	84.6%	343	15.4%	
Female	2084	85.1%	364	14.9%	
Household wealth quintiles (non pension)					0.776
1st quintile (lowest)	537	85.8%	89	14.2%	
2	733	85.4%	125	14.6%	
3	781	84.9%	139	15.1%	
4	812	83.8%	157	16.2%	
5th quintile (highest)	845	84.2%	159	15.8%	
Education level					0.001
Low	1436	87.1%	213	12.9%	
Middle	945	85.4%	162	14.6%	
High	1585	82.7%	332	17.3%	
Marital status					0.161
Not in a partnership	1235	83.8%	239	16.2%	
In a partnership (Married or cohabiting)	2730	85.4%	468	14.6%	
Ethnicity					0.201
Non-white	82	80.4%	20	19.6%	
White	3882	85.0%	686	15.0%	
Physical health (Perceived Health)					<0.001
No	3129	85.9%	513	14.1%	
Yes	836	81.2%	193	18.8%	
Mental health (CES-D depression scale)					<0.001
No depressive symptoms (<4)	3570	85.5%	605	14.5%	
Depressive symptoms (4+)	396	79.5%	102	20.5%	
ADL/IADL Difficulties					<0.001
No	3104	86.1%	502	13.9%	
Yes	862	80.8%	205	19.2%	
Difficulties walking a quarter of a mile (400 metres)					0.025
No	2929	85.6%	494	14.4%	
Yes	1034	82.9%	213	17.1%	

Chi-square test was utilized. Results were considered statistically significant at the 0.05 level (p < 0.05).

### Bivariate relationship between perceived discrimination and future falls

In Table 2, we present the relation between the frequency of perceived healthcare discrimination and falling events in wave 7 of the study to observe the bivariate association of the main exposure (frequency of healthcare discrimination) and our main outcome variable (subsequent fall). The chi-square test was employed to assess the association between the variables in the table. A significance level of *p* < 0.05 was used to determine statistical significance. With a total sample size of 3,674, the frequency of perceived healthcare discrimination is broken down into six ordinal categories that are based on participant responses, ranging from “Almost every day” to “Never.”

**Table 2.** Association of frequency of perceived healthcare discrimination and a falling event in wave 7 (n=3674).

Variable	Did not fall		Fell		p-value
	n	%	n	%	
In your day-to-day life, how often have any of the following things happened to you?					
1. Almost every day	2	66.7%	1	33.3%	0.062
2. At least once a week	6	75.0%	2	25.0%	
3. A few times a month	17	65.4%	9	34.6%	
4. A few times a year	81	69.2%	36	30.8%	
5. Less than once a year	322	76.3%	100	23.7%	
6. Never	2449	79.1%	649	20.9%	

Chi-square test was utilized. Results were considered statistically significant at the 0.05 level (p < 0.05).

Overall, these results suggest a potential trend towards a higher prevalence of falling events among those who reported perceived healthcare discrimination less frequently. It must be noted that the *p*-value is marginally not significant. This is not surprising due to the small number of observations in some of the cells and the non-parametric distribution of the responses. This Chi-square test is not meant to verify the statistical significance but the distribution and trend. A non-parametric Mann-Whitney U test was used to compare the distribution of ranks for small number of samples (table not included) just to detect the significant ordinal connection (p=0.007). This trend supports that this skewed distribution may need a transformation of the explanatory variable for analysis. Table 2 shows that two of the categories with a lower percentage of falls were number 5: “less than once a year”; and number 6 “never”. However, to ascertain the explanatory power of our cutoff point sensitivity analyses were used.

### Sensitivity analyses

The sensitivity analyses were conducted to confirm the impact of different cutoff points for measuring perceived healthcare discrimination on prospective falls in Waves 7 and 9. We used the Pearson’s Chi-square statistic, with significance set at a p-value less than 0.05 (95% Confidence Interval). The results are summarized in Table 3.

**Table 3.** Bivariate and sensitivity analyses among discrimination (wave 5) and prospective falls (Waves 7 and 9) (n=3674).

Variables	Falls at Wave 7				p-value	Falls at Wave 9				p-value
	Did not fall		Fell			Did not fall		Fell		
	n	%	n	%		n	%	n	%	
Perceived Healthcare Discrimination					0.011					0.84
No (never)	2449	79.1%	649	20.9%		1782	74.9%	598	25.1%	
Yes (almost every day, at least once a week, a few times a month, a few times a year, or less than once a year)	428	74.3%	148	25.7%		342	75.3%	112	24.7%	
Perceived Healthcare Discrimination					0.004					0.64
No (never or less than once a year)	2771	78.7%	749	21.3%		2044	74.9%	686	25.1%	
Yes (almost every day, at least once a week, a few times a month, or a few times a year)	106	68.8%	48	31.2%		80	76.9%	24	23.1%	

Chi-square test was utilized. Results were considered statistically significant at the 0.05 level (p < 0.05).

### “Never” vs “At least once” cutoff point

Our first cutoff point is at the lowest frequency of perceived discrimination (never) against all other lower frequencies. This is also the closest we could be to the mean of the measure as possible given the skewed distribution. For falls at wave 7, a significant association was observed between perceived healthcare discrimination and falls (p = 0.011). Among participants who reported “No” discrimination (never), 79.1% did not experience falls, while 20.9% experienced falls. Conversely, among those who reported “Yes” discrimination (almost every day, at least once a week, a few times a month, a few times a year, or less than once a year), 74.3% did not experience falls, and 25.7% experienced falls.

However, for falls at wave 9, no significant association was observed between discrimination and falls (p = 0.636). Among participants who reported “No” discrimination, 74.9% did not experience falls, and 25.1% experienced falls. Similarly, among those who reported “Yes” discrimination, 76.9% did not experience falls, and 23.1% experienced falls.

### “More frequent” vs “Less frequent” cutoff point

We used a different cutoff point for perceived healthcare discrimination to divide infrequent events (never and less than once a year), against more frequent events (almost every day, at least once a week, a few times a month and a few times a year). The results indicated a significant association with falls at wave 7 (p = 0.004). Among participants who reported “No” discrimination (never or less than once a year), 78.7% did not experience falls, and 21.3% experienced falls. Among those who reported “Yes” discrimination (almost every day, at least once a week, a few times a month, or a few times a year), 68.8% did not experience falls, and 31.2% experienced falls.

In terms of falls at wave 9, no significant association was found between perceived healthcare discrimination and falls (p = 0.837). Among participants who reported “No” discrimination (never or less than once a year), 74.9% did not experience falls, and 25.1% experienced falls. Similarly, among those who reported “Yes” discrimination (almost every day, at least once a week, a few times a month, or a few times a year), 75.3% did not experience falls, and 24.7% experienced falls.

This sensitivity analysis provides insights into the relationship between perceived healthcare discrimination and prospective falls. While both cutoff points are significant, the more significant influence is when the exposure group perceives discrimination more frequently. However, this common significance is only true for the relationship four years later, at wave 7. No significant relationship was observed at wave 9 for neither cutoff point.

*Multivariable association between perceived discrimination and future falls*

The multivariable logistic regression analysis in Table 4 shows that for wave 7, participants who reported frequent perceived discrimination in health care were 64% more likely to have fallen compared to those who reported less frequent discrimination (OR=1.637, 95% CI: 1.131-2.368, p=.009). Age was also a significant factor, with individuals aged 70-79 and over 80 having higher odds of falling compared to those aged 60-69 (OR=1.263, 95% CI: 1.053-1.515, p=0.012 and OR=1.59, 95% CI: 1.186-2.131, p=0.002, respectively). Females were 26% more likely to fall than males (OR=1.26, 95% CI: 1.058-1.5, p=0.01).

**Table 4.** Prospective logistic regression analyses of perceived discrimination in healthcare at baseline and subsequent falls in wave 7 and 9.

Variable	Wave 7				Wave 9			
	Participants included in the analysis (n=3454)				Participants included in the analysis (n=2664)			
	Odds Ratio	Confidence Interval 95%			Odds Ratio	Confidence Interval 95%		
	(OR)	Lower	Upper	p-value	(OR)	Lower	Upper	p-value
Perceived Discrimination in Health Care								
Less frequent (never/less than once a year) (reference)								
Frequent (almost every day to a few times a year)	1.637	1.131	2.368	0.009	0.875	0.540	1.418	0.588
Age groups								
60-69								
70-79	1.263	1.053	1.515	0.012	1.492	1.229	1.810	<0.001
Over 80	1.59	1.186	2.131	0.002	2.525	1.734	3.678	<0.001
Sex								
Male (reference)								
Female	1.26	1.058	1.5	0.01	1.099	0.910	1.327	0.325
Household wealth quintiles (non pension)								
1st quintile (lowest)(reference)								
2	0.808	0.601	1.088	0.161	0.860	0.618	1.195	0.368
3	1.015	0.758	1.361	0.919	0.859	0.617	1.197	0.370
4	0.981	0.727	1.324	0.899	0.807	0.577	1.131	0.213
5th quintile (highest)	1.182	0.871	1.603	0.284	1.011	0.722	1.415	0.950
Education level								
Low (reference)								
Middle	0.965	0.768	1.213	0.762	1.045	0.813	1.343	0.730
High	1.159	0.939	1.431	0.170	1.192	0.946	1.502	0.135
Marital status (In a partnership)								
Divorced or separated or widowed (reference)								
Married or cohabiting	0.806	0.664	0.979	0.030	0.974	0.786	1.207	0.811
Ethnicity								
Non-White (reference)								
White	1.295	0.706	2.375	0.404	1.052	0.564	1.962	0.874
Physical health (Health Self-Perception)								
Excellent/very good/good								
Fair/poor	1.238	0.984	1.559	0.069	1.190	0.910	1.556	0.203
Mental health (CES-D depression scale)								
No depressive symptoms (<4)								
Depressive symptoms (4+)	1.191	0.913	1.555	0.197	0.969	0.706	1.332	0.848
ADL/IADL difficulties								
No difficulty								
With difficulty	1.228	0.984	1.534	0.069	1.311	1.022	1.682	0.033
Walking difficulties (Difficulty walking 100 yards)								
No difficulty								
With difficulty	1.263	1.007	1.583	0.043	1.174	0.906	1.523	0.225
Constant	0.151			<.0001	-1.457			<0.001

Multivariate logistic regression analysis was performed. Results were considered statistically significant at p < 0.05.

Marital status had an impact as well, with individuals who were married or cohabiting having 20% lower odds of falling compared to those who were divorced, separated, or widowed (OR=0.806, 95% CI: 0.664-0.979, p=.03). Lastly, participants with difficulties walking a quarter of a mile (400 metres) were 26% more likely to have fallen compared to those without difficulties (OR=1.263, 95% CI: 1.007-1.583, p=0.043).

Other variables such as household wealth quintiles, education level, ethnicity, physical health, mental health, and ADL/IADL difficulties did not show significant associations with the occurrence of falls at wave 7.

For wave 9, the analysis revealed age and ADL/IADL difficulties as the significant predictors. Age remained a strong predictor, with individuals aged 70-79 and over 80 having higher odds of falling compared to those aged 60-69 (OR=1.492, 95% CI: 1.229-1.810, p<0.001 and OR=2.525, 95% CI: 1.734-3.678, p<0.001, respectively). ADL/IADL difficulties were also associated with a higher likelihood of falling (OR=1.311, 95% CI: 1.022-1.682, p=0.033).

Variables such as perceived discrimination in healthcare, sex, household wealth quintiles, education level, marital status, ethnicity, physical health, mental health, and walking difficulties did not significantly predict falls at wave 9.

## Discussion

### Interpretation of findings

Our study found that older adults who perceived discrimination in healthcare settings were significantly more likely to experience falls, adjusting for potential confounders. This observation aligns with a prior study that suggests the negative psychological and physiological impact of perceived discrimination may manifest in an increased risk of falls. This study by Reyes *et al*. ^15^ is to our knowledge the first and only study on the association of perceived discrimination and falls. They argue that that discrimination represents cumulative stress and chronic psychological trauma that add risk factors for falling. ^15^ Our results are in line with their findings on the detrimental effects of discrimination on falls.

Discrimination, according to Pascoe and Richman, is a sort of social stressor that can cause both psychological anguish and physical health issues. ^14^ In this situation, prejudice in healthcare settings may exacerbate stress and anxiety, which may in turn have an influence on physical abilities including balance and physical coordination, raising the risk of falls. According to Du Mont and Forte, ^20^ perceived prejudice was linked to worsening physical health conditions, which may be risk factors for falls or its predecessors.

The body’s stress reaction, which results in the production of stress hormones that over time can severely affect numerous bodily systems, including the musculoskeletal and neurological systems, may be directly responsible for this indirect association between perceived discrimination and falls. ^21^ This may lead to diminished muscular tone and coordination, increased frailty, and worse balance in older persons, all of which raise the risk of falls. ^9^ Previous research that has emphasised the influence of unpleasant social experiences on physical health outcomes support the stress response implicated in the link between perceived discrimination in healthcare and falls.

A conceivable mechanism that can support our findings in addition to the stress response theory is the deterioration of confidence in healthcare practitioners, which might be a result of perceived discrimination. Perceived discrimination is associated with decreased levels of confidence in healthcare practitioners, according to previous studies. ^22^^,^^23^ Lower trust can therefore lead to a person avoiding medical care, which could limit the early detection and management of fall risk factors. For instance, a person who refuses to get routine checkups because they feel discriminated against may pass up chances for eye screening, medication reviews, or balance evaluations, all of which can help prevent falls. ^9^

The experience of perceived discrimination among older adults in healthcare settings may extend beyond personal distress and mistrust; it can also manifest as disparities in the quality of healthcare provided. Discrimination can inadvertently lead to older adults being ignored, misdiagnosed, or suggested inappropriate treatments by medical staff, further exacerbating health disparities and adverse health outcomes, including increased fall risk.

Research has shown that healthcare professionals, like all individuals, can harbour implicit biases that may influence their behaviour and clinical decision-making. ^24^ These biases can subtly affect the interaction between healthcare providers and patients, leading to differential treatment and lower quality of care. ^24^ For instance, older adults who are perceived as less important due to institutional ageist biases may find their symptoms are ignored or dismissed as part of ‘normal’ aging. ^25^

Misdiagnosis is another significant concern. Studies have suggested that perceived discrimination can negatively impact patient-provider communication, ^26^ which is a crucial element in accurate diagnosis. Older persons may be less likely to completely report their symptoms or medical history if they feel discriminated against, which might result in missed diagnoses or improper treatment regimens. Perceived discrimination might potentially result in medical negligence in some severe situations. Some patients may experience lower-quality care as a result of discrimination, including the recommendation of needless or unsuitable therapies. These differences in treatment may increase the likelihood of falls among people who encounter frequent discrimination and have a direct influence on the health of older persons.

It is crucial to remember that perceived discrimination and overt abuse reflect opposite sides of the spectrum in terms of prejudice. While our study concentrated on perceived discrimination, further research is required to examine the systemic or overt types of prejudice that older persons experience in hospital settings and how these affect their risk of falling. These measurements would need to go from being perceived to being observed, becoming objective.

In line with the recognised risk factors for falls in older persons, ^9^ age and gender also had a substantial impact on the chance of falls in our research. Women and older people were more likely to fall than males and younger people. Women’s increased likelihood may be due to variables including a higher incidence of osteoporosis, which increases the risk of fractures and the fear of falling, both of which may result in less physical activity and a corresponding loss of strength and balance. ^21^ Particularly on age, since it was the only factor significant on both waves, unlike our main exposure, discrimination, that was significant only in wave 7 but not wave 9, may mean that there are latent factors linked to chronological ageing itself that may confound the effect at longer intervals.

Our research’s findings conclude that perceived discrimination in healthcare settings may increase the incidence of falls among older people. This emphasises the need of encouraging courteous, non-discriminatory healthcare environments as a potential way to lower older individuals’ risk of falling. The effectiveness of treatments aiming at reducing perceived prejudice in hospital settings, as well as potential mediating variables, should be investigated further in future studies. The lack of significance of perceived discrimination 8 years after feeling discriminated also hints to need of more detailed longitudinal studies that could try to fully grasp the conditions of older persons and discrimination continuously. Despite being a long-standing panel data, ELSA only included questions on perceived discrimination in wave 5, not allowing to see if the discriminatory acts were persistent or not. With the future inclusion of discrimination question in the upcoming wave 10 (2022-2023) we can only look forward to studies trying to cover this gap.

*Limitations*

Our study offers important new understandings into the connection between older persons’ falls and felt discrimination in healthcare settings. However, these results need to be evaluated in the context of a number of factors. First off, due to its observational methodology, our study, while identifying connections, does not demonstrate causal linkages. ^27^ The possibility of residual confounding caused by unmeasured factors cannot be totally removed even after accounting for a number of possible confounders, which means we might not be able to identify the full causal mechanism. ^27^ In this regard, it is important to emphasise that due to data limits, it was not possible to control for recognised risk factors for falls, such as impaired eyesight, the use of psychiatric medications, and polypharmacy.

Additionally, a key variable in our study, the evaluation of perceived discrimination, was assessed by self-report, which may have introduced reporting bias. ^28^ Similar to this, the occurrence of falls was self-reported, a technique that, particularly among older persons, might be subject to under-reporting and recollection bias. ^29^

While we have speculated about possible psychological and physiological pathways linking perceived discrimination to falls, the actual mechanisms were not tested directly in our study. Further research incorporating mediation analysis would be beneficial to explore these mechanisms more comprehensively. ^30^

Due to the fact that our study only included older persons (aged 60 and above), it is possible that its findings are not applicable to all population groups. Despite our best attempts to control for these ideas, factors including frailty and comorbidities that are widespread in this age group might alter both perceived discrimination and fall risk, ^31^ influencing the generalizability of our findings.

Confounding by indication is another potential concern. Those with worse health status, who are more likely to interact frequently with healthcare providers and thus may perceive more discrimination, could be at a higher risk of falls due to their underlying health status. ^32^ Furthermore, the temporal sequence of perceived discrimination and subsequent falls is not entirely clear given the design of our study. The questions for falls cover two years prior to the data collection in each wave, but there are 4 periods for the design used. Since wave 5 is the only one that asked about being discriminated, we do not know if previous or subsequent perceptions of discrimination are actually driving the falls 4 and 8 years later more strongly than our measurement at baseline.

## Conclusion

Our study, in the context of existing literature, underscores the potential influence of perceived discrimination in healthcare settings on the likelihood of falls among older adults. Our findings suggest that chronic psychological stress induced by discrimination could physiologically manifest in ways that increase the risk of falls, through both the erosion of trust in healthcare providers and potential disparities in healthcare quality. Additionally, the role of age and gender as significant factors in fall risk aligns with known determinants, hinting at an intricate interplay of demographic, social, and psychological factors shaping fall risks.

### Future research

Our work serves as a starting point for further investigation into this region, which is mostly uncharted. First, research that employs methods that can demonstrate causal links and more thoroughly rule out possible confounders is required. Future studies should take into account these elements for more thorough assessments due to the possible influence that factors like impaired vision, the use of psychiatric medications, and polypharmacy may have on fall risk.

Secondly, more nuanced approaches to measuring perceived discrimination, perhaps through the combination of self-reports with observer-rated measures, may minimize the potential for reporting bias. This could also involve broadening the scope beyond perceived discrimination to include overt forms of discrimination, aiming to create a more complete picture of the role of discrimination in healthcare settings.

Finally, the actual physiological and psychological mechanisms linking perceived discrimination to falls need further exploration. Studies employing mediation analysis could provide more insights into the underlying pathways between discrimination and fall risk.

### Recommendations

The preliminary findings of this study do suggest potential areas of intervention. As perceived discrimination in healthcare settings emerges as a potential risk factor for falls in older adults, promoting respectful and non-discriminatory environments should be considered a priority. This includes efforts to address potential implicit biases among healthcare professionals, and institutional policies promoting equality and respect for all patients.

Furthermore, strategies to improve patient-provider communication may help counter the adverse effects of perceived discrimination on diagnostic accuracy. Greater attention to the symptoms of older adults, regardless of any bias, could lead to more accurate diagnoses and appropriate treatments, potentially reducing fall risk.

Ultimately, while the relationship between perceived discrimination in healthcare settings and fall risk among older adults needs further elucidation, the findings of this study emphasize the need to ensure equality and respect in healthcare interactions, not only for their inherent ethical value but also for their potential impact on patients’ health outcomes.

#### Ethical considerations

The Multicentre Research and Ethics Committee received ethical clearance for ELSA Wave 1 data collection (Reference number MREC/01/2/91) on February 7, 2002. ^33^

The University of Tsukuba granted ethical approval for our secondary analysis (October 14, 2022, n. 1817). The present study was carried out following the Helsinki Declaration and the Ethical Guidelines for Medical and Biological Research Involving Human Subjects.

## Data Availability

English Longitudinal Study of Ageing (ELSA) http://doi.org/10.5255/UKDA-Series-200011. ^34^ This study contains the underlying data from 3 distinct waves:
• Wave 5 • Wave 7 • Wave 9 The data and relevant documentation from waves 5, 7, and 9 have been obtained and are readily available via the archive provided by the Economic and Social Data Service, which is an F1000Research-approved repository. Except for the categories of data that are considered confidential or sensitive in nature, such as linked administrative files, geographically identifiable items or genetic material information. On their website, data-archive.ac.uk, further details can be obtained via the registered DOI: 10.5255/UKDA-Series-200011. ^34^ Users just need to consent to the repository data sharing policy to have access to the data, which is publicly available.
