# Supplementary material for: The relationship of perceived discrimination in healthcare and future falls among community-dwelling older persons from an English longitudinal cohort
Source: F1000Res. 2023 Sep 11;12:1134. [Version 1] doi: 10.12688/f1000research.140302.1 (PMC10997987; doi:10.12688/f1000research.140302.1)
Supplement: Peer review history

# Reviewer report: Reviewer response for version 1

**DOI:** 10.5256/f1000research.153641.r259206

**Published:** 2024-04-05

**Authors:** Christine Waszynski

**Affiliations:** 1 Hartford Hospital, Hartford, Connecticut, USA

This article addresses two very important topics, falls and discrimination, each of which can have a profound impact upon the life of older adults .Exploring the relationship between these 2 experiences has the potential for the development of preventative and intervention strategies to improve quality of life.

 This paper uses available data from a large, longitudinal study in England known as ELSA. Self reported responses on perceived discrimination and fall episodes were analyzed and found to be associated, with those experiencing discrimination more likely to experience a fall within 4 years.

 This paper found the participants of younger age and higher educational status reported more perceived discrimination than those who were older or less educated. A discussion of this would be helpful in the article.as well as including this in a future study. 

 .

 This paper paves the way for future work that could be structured to obtain more details surrounding the population studied and their experience of discrimination. The following limitations of this study were due to the database (ELSA) utilized:

 ELSA  followed individuals for many years. This paper reported on discrimination scores collected in 2010-2011 (wave 5 ) and linked the response to falls data collected 2014-2015 (wave 7 ) and 2018-2019 (wave 9) . There was no information shared about the participants' living situation (community vs facility) at the time of the discrimination report or at the time of the fall report. This variable could have influence on both measures.

 The question regarding perceived discrimination gave choices that may not be applicable to many participants. For example, participants who could potentially report that they experience discrimination from a doctor or hospital "every day"   would need to either be hospitalized or in a care facility where they encounter a doctor every day. A better measure to use in a future study may be to ask the participant to rate the % of times they have encountered discrimination from a doctor or hospital.

 Structuring the discrimination question to describe behavior they found discriminatory rather than how often they experienced discrimination would be more informative.

 One of the discussion points in this paper was that perceived discrimination could lead to a hesitancy to seek preventative care or treatment for symptoms, therefore leading to falls. It would be useful to gather data on the frequency  of healthcare visits for those who fell vs those who did not..

 In summary, this paper reported on available data to describe the relationship between perceived discrimination and falls. It also generated opportunities for future study of these relationships.

Is the work clearly and accurately presented and does it cite the current literature?

Yes

If applicable, is the statistical analysis and its interpretation appropriate?

Yes

Are all the source data underlying the results available to ensure full reproducibility?

Yes

Is the study design appropriate and is the work technically sound?

Yes

Are the conclusions drawn adequately supported by the results?

Yes

Are sufficient details of methods and analysis provided to allow replication by others?

Yes

Reviewer Expertise:

Delirium; Gerontology

I confirm that I have read this submission and believe that I have an appropriate level of expertise to confirm that it is of an acceptable scientific standard.

# Reviewer report: Reviewer response for version 1

**DOI:** 10.5256/f1000research.153641.r235617

**Published:** 2024-02-07

**Authors:** Tszshan Ma

**Affiliations:** 1 Emory University, Atlanta, Georgia, USA

This is a thorough and clear description of an analysis of the relation of perceived healthcare discrimination and risk of falls among older adults. Overall, an excellent presentation of analyses and results, and the conclusions are supported by the data. The paper is well-written and well-discussed. Some issue of clarification has been noted below.

 Comments:

 1. This study looks at the “perceived discrimination in health care settings” as the main exposure. Based on the framing of the question, the perceived discrimination is not specifically related to age. In the manuscript, there are 4 places mentioned “perceived age discrimination” as exposure. Please correct or clarify if the discrimination is related to age. Page 6, Results – Demographic characteristics section

 Page 6, Results – Economic factors section

 Page 6, Results – Health factors section

 2. Please explain why age is categorized into 10-year bins in the regression model instead of treating it as a continuous variable.

 3. Among adjusted confounders, age, difficulties walking, perceived physical health, and ADL/IADL difficulties are both significantly/marginally significantly associated with exposure and outcome. It is suggested to perform additional effect modification analyses to assess whether these characteristics could modify the relation between exposure and outcome. This could provide potential insights in identifying subgroups of persons who are more susceptible to the effect of perceived discrimination on risk of falls, hence delineating potential pathways linking perceived discrimination to falls.

Is the work clearly and accurately presented and does it cite the current literature?

Yes

If applicable, is the statistical analysis and its interpretation appropriate?

Yes

Are all the source data underlying the results available to ensure full reproducibility?

Yes

Is the study design appropriate and is the work technically sound?

Yes

Are the conclusions drawn adequately supported by the results?

Yes

Are sufficient details of methods and analysis provided to allow replication by others?

Yes

Reviewer Expertise:

Epidemiologist

I confirm that I have read this submission and believe that I have an appropriate level of expertise to confirm that it is of an acceptable scientific standard.
